# The BioFIND study: Characteristics of a clinically typical Parkinson's disease biomarker cohort

**DOI:** 10.1002/mds.26613

**Published:** 2016-04-26

**Authors:** Un Jung Kang, Jennifer G. Goldman, Roy N. Alcalay, Tao Xie, Paul Tuite, Claire Henchcliffe, Penelope Hogarth, Amy W. Amara, Samuel Frank, Alice Rudolph, Cynthia Casaceli, Howard Andrews, Katrina Gwinn, Margaret Sutherland, Catherine Kopil, Lona Vincent, Mark Frasier

**Affiliations:** ^1^ Division of Movement Disorders, Department of NeurologyColumbia University Medical CenterNew YorkNew YorkUSA; ^2^ Section of Parkinson Disease and Movement Disorders, Department of Neurological SciencesRush University Medical CenterChicagoIllinoisUSA; ^3^ Parkinson Disease and Movement Disorder Program, Department of NeurologyUniversity of ChicagoChicagoIllinoisUSA; ^4^ Department of NeurologyUniversity of MinnesotaMinneapolisMinnesotaUSA; ^5^ Department of NeurologyWeill Cornell Medical CollegeNew YorkNew YorkUSA; ^6^ Department of Molecular and Medical GeneticsOregon Health & Science UniversityPortlandOregonUSA; ^7^ Department of NeurologyUniversity of Alabama at BirminghamBirminghamAlabamaUSA; ^8^ Department of NeurologyBeth Israel Deaconess Medical CenterBostonMassachusettsUSA; ^9^ Center for Human Experimental Therapeutics, Clinical Trials Coordination CenterUniversity of RochesterRochesterNew YorkUSA; ^10^ National Institute of Neurological Disorders and StrokeNational Institutes of HealthBethesdaMarylandUSA; ^11^ The Michael J. Fox Foundation for Parkinson's ResearchNew YorkNew YorkUSA

**Keywords:** biomarkers, cerebrospinal fluid, DNA, plasma, RNA, saliva, urine

## Abstract

**Background:**

Identifying PD‐specific biomarkers in biofluids will greatly aid in diagnosis, monitoring progression, and therapeutic interventions. PD biomarkers have been limited by poor discriminatory power, partly driven by heterogeneity of the disease, variability of collection protocols, and focus on de novo, unmedicated patients. Thus, a platform for biomarker discovery and validation in well‐characterized, clinically typical, moderate to advanced PD cohorts is critically needed.

**Methods:**

BioFIND (Fox Investigation for New Discovery of Biomarkers in Parkinson's Disease) is a cross‐sectional, multicenter biomarker study that established a repository of clinical data, blood, DNA, RNA, CSF, saliva, and urine samples from 118 moderate to advanced PD and 88 healthy control subjects. Inclusion criteria were designed to maximize diagnostic specificity by selecting participants with clinically typical PD symptoms, and clinical data and biospecimen collection utilized standardized procedures to minimize variability across sites.

**Results:**

We present the study methodology and data on the cohort's clinical characteristics. Motor scores and biospecimen samples including plasma are available for practically defined *off* and *on* states and thus enable testing the effects of PD medications on biomarkers. Other biospecimens are available from *off* state PD assessments and from controls.

**Conclusion:**

Our cohort provides a valuable resource for biomarker discovery and validation in PD. Clinical data and biospecimens, available through The Michael J. Fox Foundation for Parkinson's Research and the National Institute of Neurological Disorders and Stroke, can serve as a platform for discovering biomarkers in clinically typical PD and comparisons across PD's broad and heterogeneous spectrum. © 2016 The Authors. Movement Disorders published by Wiley Periodicals, Inc. on behalf of International Parkinson and Movement Disorder Society

The diagnosis of Parkinson's disease (PD) currently relies upon clinical criteria.^1^ No existing laboratory test diagnoses PD or gauges the effectiveness of a treatment on underlying disease processes. Molecular imaging techniques are helpful for diagnosis, but are costly and cannot distinguish among different parkinsonian syndromes.^2^, ^3^ Current methods for diagnosing, treating, and prognosticating PD are inadequate and would be greatly improved by the discovery and validation of biomarkers, namely, those “characteristics that are objectively measured and evaluated as indicators of normal biologic processes, pathogenic processes, or pharmacologic response to a therapeutic intervention.”^4^

To date, no single biomarker demonstrates optimal utility in diagnosis, disease progression, and therapeutic monitoring. Different biomarkers may be needed, individually or in combination, to address these questions. In addition, biomarker studies reveal a striking degree of variability of data not only within PD, but also when comparing PD to controls.^5^, ^6^ Differences in biomarker sample collection and processing may contribute to this variability,^7^ which may be further compounded by the heterogeneity of PD. Currently, such variability hinders the use of biomarkers for individual diagnosis or therapeutic monitoring. For example, the Parkinson's Progression Markers Initiative (PPMI)^8^ focuses on early, untreated PD subjects, and although it utilizes dopamine transporter imaging (DaTSCAN) to identify subjects with dopaminergic deficits, the cohort may likely include other parkinsonian syndromes that are indistinguishable from PD at that early stage.^9^ Thus, discovery and validation of biomarkers in a well‐characterized, “typical” (including medically treated and therefore more advanced than the de novo, untreated cohorts) PD population is a critical complementary step for advancing biomarker discovery in PD. The BioFIND (Fox Investigation for New Discovery of Biomarkers in Parkinson's Disease) study addressed these needs by (1) establishing a cohort of moderate to advanced PD patients, typical of those PD who would most likely be encountered in the clinical practice or a trial setting^10^, ^11^, ^12^ (Fig. 1) and (2) developing a repository of standardized, rigorously collected clinical data and biospecimens for biomarker research. BioFIND resources can therefore be used as an infrastructure for biomarker discovery in PD, comparing clinically typical PD to healthy controls (HCs), and subsequently foster comparisons of moderate to advanced or treated PD across the broad and heterogeneous landscape of PD, including de novo, untreated PD. This article describes the design, eligibility criteria and rationale, and data and sample collections of BioFIND and presents data on the cohort characteristics.

**Figure 1:**
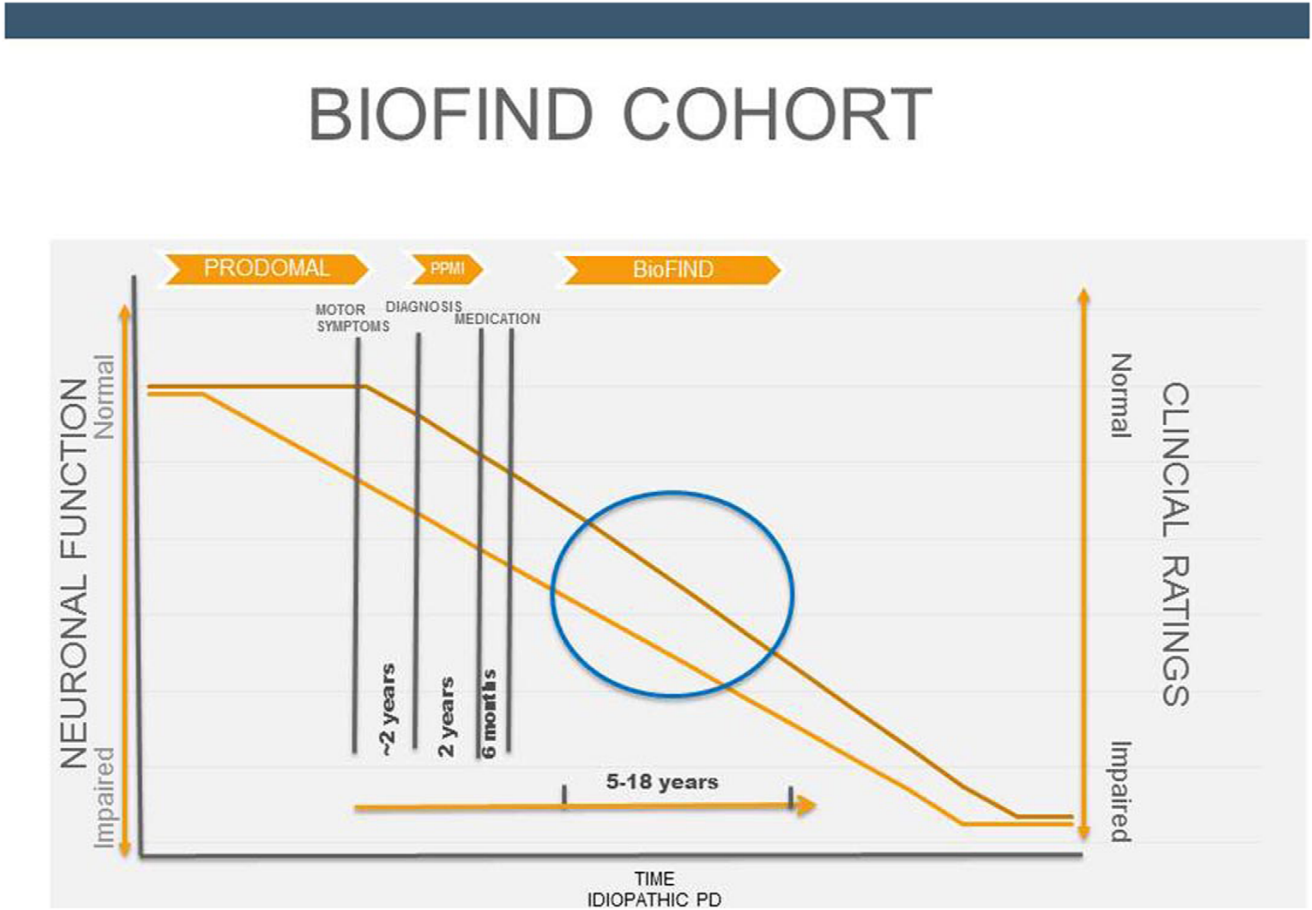
BioFIND cohort in the context of other biomarker studies in PD. Darker line (brown) represents progression of clinical rating, and lighter line (orange) shows hypothesized progression of neuronal function that precedes the motor manifestation. BioFIND included PD patients who have 5 to 18 years of PD from motor symptom onset. This complements PPMI subjects who have less than 2 years of PD from the time of diagnosis and are expected to not require medications for additional 6 months. In both BioFIND and PPMI, the average interval from symptom onset to diagnosis is around 2 years.

**Figure 2:**
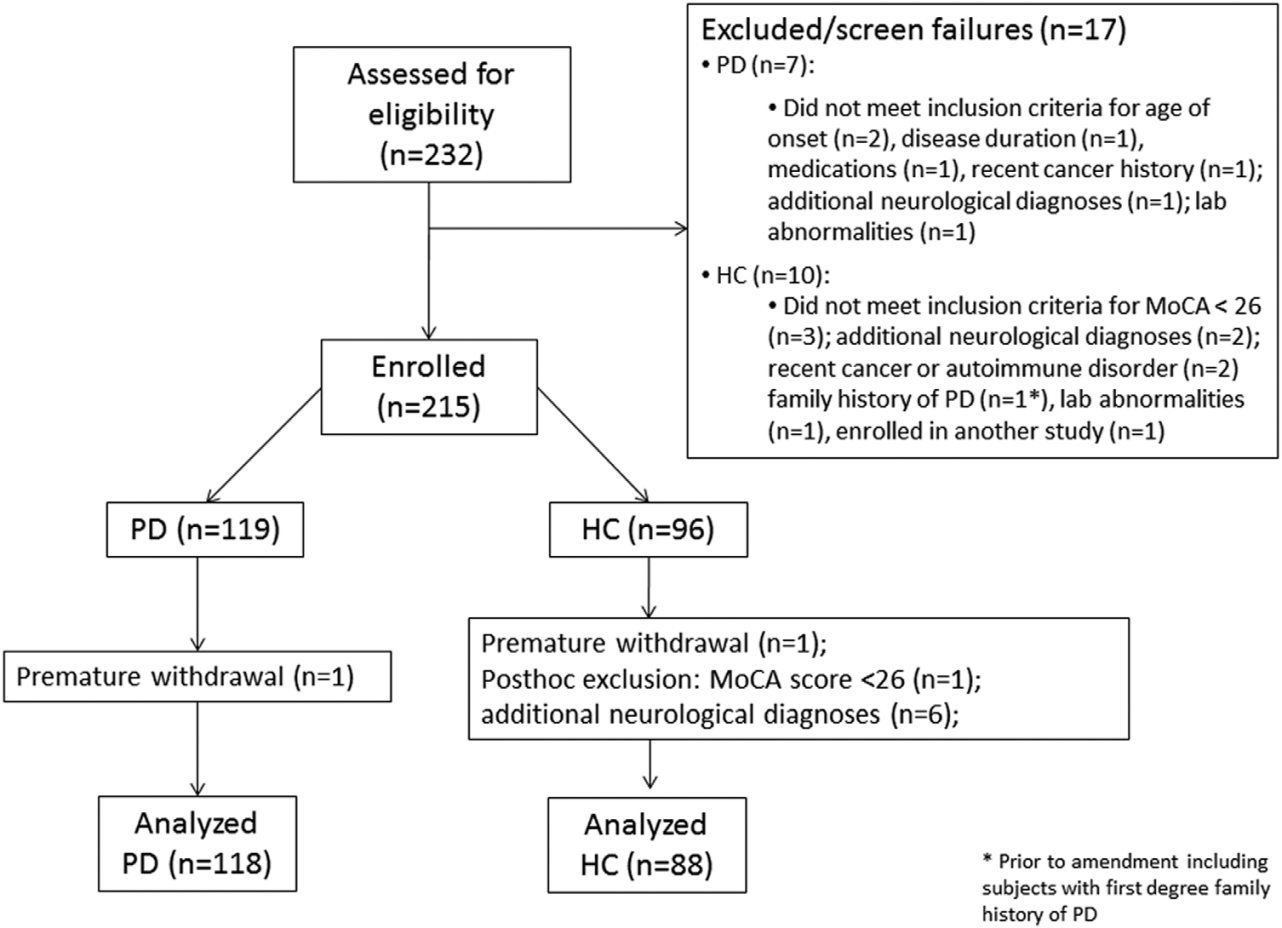
Enrollment and selection of subjects. We excluded 7 PD subjects who did not meet the inclusion criteria of age of onset, disease duration, additional neurological diagnoses, or abnormal labs and 10 HC subjects who had low MoCA scores (<26), other neurological diagnoses (including 3 with tremor and 3 with neuropathies), or abnormal labs. An additional 7 HC subjects were excluded from the analyses of cohort characteristics after enrollment for similar reasons. One subject in each category did not complete the study.

## Study Design and Rationale

BioFIND is a cross‐sectional, observational study of moderate to advanced PD subjects and HCs evaluated with standardized clinical and biospecimen acquisition protocols. Participants were enrolled at eight sites in the United States with expertise in PD subject recruitment, assessment, and biospecimen collection. BioFIND carefully standardized study procedures to minimize preanalytical variability associated with sample processing and utilized the same procedures, wherever possible, as those used in PPMI, thereby enhancing further cross‐study comparisons.^8^

### PD Inclusion Criteria

#### PD Diagnostic Criteria and Motor and Cognitive Features

Enrolled PD subjects met the United Kingdom PD Society Brain Bank (UKPDBB) clinical diagnostic criteria, modified to require all three classic motor signs of parkinsonism (i.e., bradykinesia, rigidity, and resting tremor), by history or examination, instead of just two signs (bradykinesia with either rigidity, resting tremor, or postural instability).^13^, ^14^, ^15^ The modified eligibility criteria were employed to increase diagnostic specificity of PD and were based upon several factors. These include PPMI database analyses of 447 cases with deficits on DaTSCAN and 81 scans without evidence of dopaminergic deficit (accessed in July 2014), which found that those with all three motor signs demonstrated a 92% chance of an abnormal DaTSCAN, but only a 72% chance when just two motor signs were observed (*P* < 0.001). Further influencing our inclusion criteria is evidence from a published series of case studies from pathologically confirmed PD patients, in which resting tremor and asymmetry at onset were present in 68% and 70% of patients, respectively.^14^, ^15^ Unilateral onset and/or persistence of asymmetric symptoms were required and are supportive positive features of the UKPDBB criteria, particularly when persisting for longer than 3 years.^13^, ^16^ Notably, whereas resting tremor may be present in atypical parkinsonian syndromes, it is less common than observed in PD.^17^ BioFIND included all H & Y stages. Because prevalence estimates of mild cognitive impairment or dementia in moderate to advanced PD are 25% to 40%, we included PD subjects regardless of cognitive status as assessed by their Montreal Cognitive Assessment (MoCA) score.^18^, ^19^

#### PD duration

Disease duration, defined as time since motor symptom onset, in enrolled subjects ranged from 5 to 18 years. We found no significant difference in the interval from estimated disease onset to time of diagnosis in BioFIND PD subjects compared with 447 subjects in PPMI, which used time of diagnosis as the inclusion criteria (average, 1.8 and 1.5 years, respectively; *P* > 0.05).

We chose a disease duration of 5 years or greater to improve diagnostic accuracy given that mild or subtle findings of early PD may overlap with other neurodegenerative diseases and confound diagnosis. As demonstrated in a clinicopathological study, only 26% of subjects were accurately diagnosed clinically if untreated or not clearly responsive to dopaminergic agents; 53% were accurately diagnosed with less than 5 years of disease duration and response to medications; however, there was greater than 85% diagnostic accuracy in PD subjects with 5 or more years of disease and medication response.^9^

#### PD Onset Age and Genetics

Twin studies show that genetics impact PD risk when onset precedes 50 years of age,^20^ and cohort studies demonstrate that a positive first‐degree family history of PD is associated with carrying a PD‐related genetic variant.^21^ Approximately 30% of Ashkenazi Jews (AJs) carry either leucine‐rich repeat kinase (*LRRK2)* or glucocerebrosidase (*GBA*) variants.^21^, ^22^ To minimize recruitment of individuals with genetic PD (younger) or unrelated comorbidities (older), study inclusion was limited to ages 50 to 75 years at disease onset (with resultant age at enrollment of 55‐93 years). PD cases with a first‐degree family history and all AJ participants were initially excluded from the study for the same reason, but were later included after the steering committee recommended genotyping all subject samples after study enrollment. The NeuroX array (Illumina; San Diego, CA) was used to genotype all samples.^23^, ^24^

#### Medication response

PD subjects were required to have a well‐established response to one or more dopaminergic agents and/or amantadine given that this supports the diagnosis of PD.^13^ Medication response was determined using best clinical judgment of the movement disorder clinician based upon historical report by the patient and/or direct observation. Though some have advocated an acute levodopa challenge,^25^ we did not require this because prominent placebo effects can occur in the short term. Rather, long‐term response to l‐dopa, as evidenced by patient report or clinician judgment, is more reliable.^26^, ^27^

### PD Exclusion Criteria

We excluded subjects who had any of the following signs or symptoms, as judged by the clinician, that are suggestive of atypical or secondary parkinsonian syndromes, such as MSA, PSP, corticobasal ganglionic degeneration, vascular or postencephalitic parkinsonism: early severe autonomic dysfunction; cerebellar signs; supranuclear palsy; corticospinal tract signs; or lower‐body predominance.

Other exclusion criteria included a history of cancer (except basal or squamous cell skin cancers) within 5 years preceding enrollment, autoimmune disorders, liver disease, or hematological disorders to minimize confounding markers in blood or cerebrospinal fluid (CSF). Treatments with anticoagulants, conditions precluding the safe performance of the lumbar puncture, use of investigational drugs or devices, or significant lab abnormalities were also exclusionary. Those with ablative brain surgery or DBS were excluded based on study findings of altered protein expression in CSF in PD patients post‐DBS implantation.^28^

### Healthy Control Inclusion and Exclusion Criteria

HCs were group‐matched by age and sex to PD subjects with similar age inclusion criteria of 55 to 93 years. Controls were free of any known neurological disorders and scored ≥26 on the MoCA. This cut‐off score, also used in PPMI, has 90% sensitivity and 87% specificity in detecting MCI in non‐PD populations.^18^ Other exclusion criteria for controls were similar to those for PD subjects, as outlined above. Controls were excluded if they had a first‐degree family member with PD.

### Subject Recruitment

BioFIND enrollment was initiated in December 2012 and completed in June 2015. Novel recruitment strategies included patient‐oriented events throughout the United States sponsored by The Michael J Fox Foundation (MJFF), use of the MJFF Fox Trial Finder (https://foxtrialfinder.michaeljfox.org), and geotargeted social media and blog posts by participants sharing their research experience (Supporting Table S1). Each site recruited participants by PD support groups, PD or control registries, or clinic encounters with PD patients and their spouses, unrelated care partners, and other individuals who served as controls. All study protocols and recruitment strategies were approved by the institutional review boards for the University of Rochester Clinical Trials Coordination Center (CTCC) and individual sites.

### Data and Biospecimen Collection

Subjects underwent standardized study assessments for clinical data, and biospecimens were collected in a standardized fashion at two visits: baseline (V1) and follow‐up within 2 weeks of baseline (V2). For PD subjects, V1 was performed in the *on* state (1‐3 hours after the last PD medication dose) and V2 was performed in the practically defined *off* state (early morning before PD medications approximately 12 hours after the last dose the night before).^29^ Specimens were collected in both *on* and *off* states because few studies have been able to evaluate the acute medication effects on biospecimens. V1 included collection of blood for DNA and plasma and clinical assessments including the International Parkinson and Movement Disorder Society–Sponsored revision of the UPDRS (MDS‐UPDRS) parts I to IV for PD subjects and part III only for control subjects.^30^ V2 included collection of blood for RNA and plasma, lumbar puncture for CSF collection in all subjects, and MDS‐UPDRS part III in PD subjects. All PD and control subjects either fasted or, if unable, had a low‐fat diet on the morning of V2. Collection of saliva and urine samples at V2 was added after study startup, and these specimens are available for around 25% of the total BioFIND cohort (Table 3).

Clinical data included demographics, family history of PD, medical/neurological histories, and medications. Assessments included neurological exam, the MDS‐UPDRS,^30^ MoCA,^18^ and Rapid Eye Movement (REM) Behavior Sleep Disorder Questionnaire (RBDSQ)^31^ and for PD subjects, the Modified Schwab and England Activities of Daily Living Scale.^32^ These clinical assessments are also used in PPMI^8^ and the National Institute of Neurological Disorders and Stroke (NINDS) Parkinson's Disease Biomarkers Program (PDBP)^33^ and thus provide continuity for examining biomarkers across the PD spectrum and across multiple clinical sites.

Multiple biofluids, including blood for DNA, RNA, plasma, CSF, saliva, and urine, were collected and banked as recent data supports their potential in PD biomarker discovery.^6^, ^34^, ^35^, ^36^, ^37^, ^38^ Local laboratories at each clinical site analyzed blood at V1 for prothrombin/partial thromboplastin time (PT/PTT) and complete blood count (CBC) to determine safety of performing a lumbar puncture; at V2, local laboratories measured glucose, protein, and cell counts in CSF. Processing, storage, and timing of sample collection are noted in Table 1. To ensure standardization, the NINDS Repository provided all specimen collection kits, coordinated clinical site training on biospecimen collection protocols, performed centralized biospecimen quality assessment, extraction, banking, subaliquoting, and distribution. Detailed and standardized biospecimen collection, processing, and shipping (e.g., volume, aliquoting methods, centrifuge speeds and times, and so on) ensured the highest quality and uniformity of preanalytical variables in the sample collection. Additional details are available in the BioFIND Laboratory Manual (Supporting Information), and laboratory case report form data are available through the BioFIND database repository.

**Table 1:** BioFIND specimen sample collection and processing, V1 and V2

Sample Type	Tube Type	No. of Tubes Supplied in Kit	Processing/Aliquoting	Tubes Shipped to Biorepository
Whole blood: for PT/PTT analysis at individual site lab	2.7‐mL light blue top sodium citrate tube	1	NA	0
Whole blood: for CBC, platelets analysis at individual site lab	10‐mL Lavender Top EDTA Tube	1	NA	0
Whole blood: for isolation of plasma/pellet	2‐mL microcentrifuge tubes	6	1‐mL plasma aliquots in each 2‐mL microcentrifuge tube	3
	10‐mL lavender top EDTA Tube	1	Retain blood pellet in EDTA tube	0
Whole blood: for extraction of DNA	8.5‐mL yellow top ACD tube	1	NA	1
Total V1		10		4
Whole blood: for RNA extraction	2.5‐mL PAXgene tube	3	NA	2
Whole blood: for isolation of plasma/pellet	2‐mL microcentrifuge tubes	18	1‐mL plasma aliquots in each 2‐mL microcentrifuge tube	9
	10‐mL lavender top EDTA tube	3	Retain blood pellet in EDTA tube	2
CSF	50‐mL conical tube	1	Combine and mix total CSF	NA
	15‐mL conical tubes	2	Divide and spin total CSF	NA
	2‐mL microcentrifuge tubes	18	1‐mL CSF aliquots in each 2‐mL microcentrifuge tube	10
	2‐mL purple‐top microcentrifuge tubes	2	1‐mL CSF aliquots in each 2‐mL purple‐top microcentrifuge tube	0
Saliva	50‐mL conical tube	1	Processed and centrifuged in 15‐mL conical tubes; aliquoted in 2‐mL microcentrifuge tubes	6
	15‐mL conical tube	2		
	2‐mL microcentrifuge tubes	10		
Urine	50‐mL orange top cup	1	Centrifuged and aliquoted in 15‐mL conical tubes	1
	15‐mL conical tubes	4		
Total V2		65		30

ACD, acid citrate dextrose; EDTA, ethylenediaminetetraacetic acid; NA, not applicable; PT/PTT, prothrombin/partial thromboplastin time.

### Standardization of Clinical Data Acquisition

Standardized methods for acquisition of study data included: CTCC designing standardized worksheets for recorded data, data entry into the CTCC's web‐based electronic data capture application (eClinical), range checks at the time of data entry, frequent cross‐form and logic checks on the database, and queries or data corrections made by sites in the electronic application. Additional data quality checks and reconciliation were provided by Columbia University Data Coordinating Center. Study manuals outlined proper collection of clinical and biospecimen data. Study documents were shared with sites by the CTCC's secure web‐portal. Before enrolling subjects, site study staff were trained in evaluating subjects (e.g., MDS‐UPDRS) and electronic data capture.

#### Specimen and Data Access

The Bioinformatics Core at the Laboratory of Neuroimaging at University of Southern California curates and distributes the BioFIND's clinical and biospecimen data (http://biofind.loni.usc.edu/). To ensure biospecimen quality, the NINDS Repository performed quality control assessments. Purity of DNA extracted from fresh whole blood at the Repository was assessed by spectrophotometric analysis of 260/280 absorbance. Fluorescent polymerase chain reaction microsatellite analysis was performed on extracted DNA to determine sample identity and verify sex against clinical data and confirm subject sample consistency across both study visits. The quality of RNA extracted from frozen PAXgene tubes at the Repository was assessed by several metrics, including analysis of 260/280 and 260/230 absorbance and RNA integrity number (RIN; Agilent TapeStation 2200 Bioanalyzer; Agilent Technologies, Santa Clara, CA). To assess for blood contamination, CSF hemoglobin (Hb) levels were measured using a human Hb enzyme‐linked immunosorbent assay quantitation (Bethyl Laboratories, Montgomery, TX). Plasma Hb data (BioAssay Systems LLC, Hayward, CA) are also available in the BIoFIND database.

Information for accessing BioFIND study documents, clinical data, and biospecimens can be found through the MJFF website (https://www.michaeljfox.org/biofind) and National Institutes of Health (NIH) X01 mechanism (http://grants.nih.gov/grants/guide/pa‐files/PAR‐14‐340.html).

### Statistical Analysis

The BioFIND study aimed to develop a data and sample repository for future biomarker studies and thus did not include a predefined power calculation. Baseline characteristics of PD and HC groups were compared using two‐tailed *t* test and chi‐square tests, with SPSS statistical software (v23; SPSS, Inc., Chicago, IL). Test scores, age, and other continuous measures were analyzed using parametric tests (*t* tests) because the distributions of these variables were generally symmetrical, and because the sample sizes were sufficiently large that these tests can be considered robust with respect to potential departures from normality; for individual tests, in the case of significant differences between groups in variance, the standard error of the mean and significance tests were based on well‐established statistics for analysis of groups with unequal variance.

## Results

The BioFIND study enrolled 119 PD and 96 HC subjects, of whom 118 PD and 88 HC subjects were included in final analyses. Figure 2 depicts the flow diagram of enrollment to final analysis. Data on the clinical characteristics of the cohort are summarized in Table 2. Mean age at enrollment of PD subjects was 68 years and 63% were males; PD participants were slightly older and included more males compared to HCs, but these differences were not statistically significant. Educational levels and ethnic and race compositions were similar in both subject groups. Mean duration from onset of PD symptoms to enrollment was 8.5 years (standard deviation [SD]: 3.2). Mean total MDS‐UPDRS part III score at V2 (*off* state) was 39.1 (SD, 13.1) compared with PPMI's (untreated at baseline) mean of 20.9 (SD, 4.5), reflecting the inclusion of more advanced patients in BioFIND.

**Table 2:** Cohort demographics and clinical characteristics

	PD	HC	*P* Value
Age, years	68.0 (6.5)	65.6 (7.4)	0.015
Male, %	62.7	51.1	0.12
Weight, kg	80.8 (18.1)	79.1 (17.8)	0.49
Ethnicity, % Hispanic	3.4	5.7	0.50
Race, % white	93.2	89.9	0.45
Education, years	17.0 (3.0)	17.0 (3.1)	1.0
Disease duration, years	8.5 (3.2)	NA	
MoCA	26.8 (2.5)	27.8 (1.4)	<0.001
RBD, % (questionnaire score ≥5)	52.5	12.5	<0.001
MDS‐UPDRS Part I	9.4 (5.6)	NA	
MDS‐UPDRS Part II	11.0 (6.3)	NA	
MDS‐UPDRS Part III–ON (V1)	28.6 (13.7)	1.6 (2.0)	<0.001
MDS‐UPDRS Part III–OFF (V2)	39.1 (13.1)	NA	
MDS‐UPDRS total (I–III)‐–ON	49.0 (20.6)	NA	
H & Y stage–ON	2.0 (0.58)	0.0 (0.0)	<0.001
H & Y stage–OFF	2.2 (0.67)	NA	
TD subtype proportion, %	33.3	NA	
PIGD subtype, %	62.3		
Intermediate subtypes, %	4.4		

Values reported as mean (SD), unless otherwise noted; *P* values are for t tests or chi‐square for categorical variables (statistics given in %).

A unique feature of the BioFIND cohort is the collection of plasma specimens during both *off* and *on* states. Mean MDS‐UPDRS part III scores were 10.5 points lower (representing a 27% improvement) at V1 (*on*) compared to V2 (*off*), which is similar to clinically important differences cited in other studies.^39^ Almost all items in MDS‐UPDRS part III motor scores were milder in the PD subjects at V1 compared to V2, likely reflecting medication effects in the *on* state. Although we required the presence of tremor, all motor subtypes were represented as follows: tremor dominant (TD), postural instability and gait disorder (PIGD), and intermediate at 45.8%, 39.0%, and 15.3%, respectively, at V1 per criteria defined by Jankovic and colleagues^40^ and adapted by Stebbins and colleagues.^41^ PD motor subtypes, calculated at V2 using MDS‐UPDRS part III motor scores from V2 (*off*) plus the subjective history questions from MDS‐UPDRS part II from V1 (acquired only once during the study) showed slightly different proportions from V1: TD, PIGD, and intermediate at 51.7%, 36.4%, and 11.9%, respectively. These differences indicate that subtype classification can vary with motor state and medication effects.

Mean MoCA score in PD subjects (26.8; SD, 2.6) was significantly lower than in HCs, which was expected given that our HC exclusion criteria of MoCA ≤26. MoCA subscores that differed significantly in PD from HC were delayed recall (3.5 vs. 4.0; *P* = 0.004) and orientation (5.9 vs. 6.0; *P* = 0.018). REM sleep behavior disorder (RBD) defined by RBDSQ score greater than or equal 5 occurred in 53% of PD and 13% of HC subjects. Although weight loss has been noted in PD patients,^42^ body weight did not significantly differ between subject groups.

Available biospecimens are summarized in Table 3. Overall rate of clinical site adherence to sample collection and processing protocols was high. For example, a >30‐minute delay in time to sample freezing beyond the 60‐minute guideline was noted only in 1.4% of samples collected. DNA 260/280 absorbance had a median of 1.83 and range of 1.74 to 1.93 for the 222 samples, suggesting a high DNA purity. Of the 415 RNA samples from 212 unique subjects, median RIN value was 7.1, with <2% of samples having low quality (RIN <3) and 85% having high quality (RIN ≥6). Of 193 unique CSF samples, 66% had Hb levels below the assay limit of detection, 15% within the range of 30 to 200 ng/mL, and 19% >200 ng/mL indicative of blood contamination.

**Table 3:** Summary of biospecimens available

Available Biospecimen From Unique Subjects								
Subjects	DNA	Plasma (V1)	Plasma (V2)	Whole Blood Pellet	RNA	CSF	Saliva	Urine
PD (included in analyses)	117	118	117	115	118	109	23	27
HC (included in analyses)	88	88	87	87	87	78	26	27
Excluded (at screening)	8	8	—	—	—	—	—	—
Withdrawn	2	2	—	—	—	—	—	—
Excluded (post hoc)	7	8	7	7	7	6	1	1
Totals	222	224	211	209	212^a^	193	50	55

a Two unique RNA samples available for 203 of 212 subjects.

## Discussion

The BioFIND study provides a unique, valuable resource for discovery and validation of PD biomarkers. Strengths of the BioFIND cohort include enrollment of a carefully defined and characterized PD cohort with “typical or classic” moderate to advanced disease, with high confidence of clinical diagnostic accuracy based upon carefully chosen inclusion/exclusion criteria. Presence of the core clinical signs outlined above for PD inclusion, along with the absence of atypical features detailed here, yields a positive predictive value of greater than 95% for the diagnosis of idiopathic PD.^43^ Studies of biochemical analytes in biospecimens from the BioFIND repository can serve as a benchmark for detecting meaningful, reliable differences between PD and HCs. Additional strengths of BioFIND include standardized clinical data and biospecimen collection with rigorous, uniform procedures as well as overlapping data elements with the PPMI and NINDS PDBP study. Furthermore, *on* and *off* medication assessments and plasma collection in PD subjects will allow for comparisons of assays in different motor states and dopaminergic medication‐induced short‐term changes, although long‐term changes attributed to medication are not likely to be distinguished by this comparison. Some biofluid‐based biomarkers have been shown to be different in early de novo patients compared with more advanced, medicated PD patients treated.^44^, ^45^ However, the reasons for this are not clear and may relate to disease duration or severity. BioFIND's design uniquely allows researchers to distinguish the acute effect of medications from disease severity on such biomarkers. Moreover, BioFIND may contribute to discovery and validation of much needed biomarkers for disabling nonmotor PD symptoms, including sleep and cognitive dysfunction, because of its comprehensive clinical data set.

Comparison of this representative, homogeneous PD cohort with HCs enhances the likelihood of biomarker discovery and will provide a first step in discovery of PD‐associated markers. HC subjects were recruited from various sources and included only individuals without a first‐degree family history of PD or potentially confounding neurological or medical conditions. It is likely, however, that these controls may not accurately reflect the general population, being “supernormal,” that is, lacking the typical prevalence of various conditions found in a population.^46^ The rationale for use of such controls is to increase the chance of finding differences between PD subjects and controls while minimizing variability. Promising and positive findings in the studies utilizing the BioFIND samples can subsequently be replicated in larger patient groups or cohorts with more heterogeneous HC features and may guide future research directions to determine whether differences observed between PD and HCs are truly related to PD‐associated factors.

BioFIND, along with other observational cohorts focused on building resources for biomarker studies (e.g., PPMI and PDBP),^8^, ^33^ overlaps in several clinical assessments and biospecimens collected and thereby allow for comparison across cohorts. Furthermore, BioFIND harmonizes with the PPMI protocols whenever possible and thus provides continuity across the disease spectrum from de novo, unmedicated to advanced PD patients. BioFIND biospecimens have the potential to transform our understanding of PD and biomarkers of the disease. We anticipate that BioFIND, particularly within the landscape of other recent biomarker efforts, will enable rapid and seamless progress in the field of PD biomarkers as we move from the discovery phase into the validation phase and, subsequently, into clinical use.

## Author Roles

(1) Research Project: A. Conception, B. Organization, C. Execution; (2) Statistical Analysis: A. Design, B. Execution, C. Review and Critique; (3) Manuscript: A. Writing of the First Draft, B. Review and Critique.

U.J.K.: 1A, 1B, 1C, 2A, 2B, 2C; 3A, 3B

J.G.G.: 1A, 1B, 1C, 3A, 3B

R.A.: 1A, 1B, 1C, 3A, 3B

T.X.: 1A, 1B, 1C, 3A, 3B

P.T.: 1A, 1B, 1C, 3A, 3B

C.H.: 1A, 1B, 1C, 3A, 3B

P.H.: 1B, 1C, 3A, 3B

A.W.A.: 1B, 1C, 3A, 3B

S.F.: 1B, 1C, 3A, 3B

A.R.: 1B, 1C, 3A, 3B

C.C.: 1B, 1C, 3B

H.A.: 1C, 2B, 2C, 3B

K.G.: 1B, 1C, 3A, 3B

M.S.: 1B, 1C, 3B

C.K.: 1B, 1C, 3A, 3B

L.V.: 1B, 1C

M.F.: 1A, 1B, 1C

## Financial Disclosures

U.J.K. has served on the advisory boards of APDA, PDF, Caremark/CVS; has received honoraria from NIH study section, Parkinson's Disease Foundation (PDF) grant reviews, and State University of New York; has received grant support from NIH, MJFF, and PDF; and has been employed by Columbia University Medical Center. J.G.G. has held a consultancy with Acadia, Pfizer, and Teva has received honoraria from MDS, and the American Academy of Neurology; has received grant support from NIH, MJFF, PDF, Rush University, Acadia, Teva (Moderato study, site principal investigator [PI]), and Biotie (Synapse, site PI); and has been employed by Rush University Medical Center. R.A. has held consultancies with Genzyme/Sanofi and Prophase; has received grant support from NIH, MJFF, PDF, and the Smart Foundation; and has been employed by Columbia University Medical Center. T.X. has held a consultancy with Caremark/CVS; has received grant support from NIH, MJFF, GE Healthcare, and University of Chicago; and has been employed by University of Chicago. P.T. has received grant support from NIH, MJFF, Parkinson UK, Kyowa, and General Electric; has been employed by University of Minnesota; and has received royalties from Cambridge Press. C.H. has held a consultancy with Pfizer; has served on the advisory boards of Acadia, IMPAX, and Teva; has received honoraria from American Academy of Neurology, American Society for Neuroimaging, Davis Phinney Foundation, and Neuroalert; has received grant support from NINDS, NYSTEM, Biogen, MJFF, PDF, CV Starr Foundation, Solomon Family Foundation, the Dana Foundation; has been employed by Weill Cornell Medical College; and has served on the speaker's bureaus for Acadia, GE Healthcare, IMPAX, and Teva. P.H. has received honoraria from the Korean Movement Disorders Society; has received grant support from MJFF, Vertex Pharmaceuticals, Inc, and European Commission 7th Framework Programme on Research (TIRCON); has been employed by Oregon Health & Science University; and has received salary support from an unrestricted donation to the OHSU Foundation from Retrophin, Inc. A.W.A. has received honoraria from the American Academy of Neurology; has received grant support from NIH; and has been employed by University of Alabama at Birmingham. S.F. has received honoraria from the Johns Hopkins Dystonia and Spasticity course; has received grant support from Auspex Pharmaceuticals, MJFF, and APDA; and has been employed by Boston University. C.J.C. has received grant support from MJFF and has been employed by University of Rochester Medical Center. K.G. has been employed by NIH. M.S. has been employed by NIH. C.M.K. has been employed by MJFF. L.V. has been employed by MJFF. M.A.F. has served on the Alzheimer's Disease Initiative (ADNI) Scientific Advisory Board and has been employed by MJFF.

## Supporting information

Additional supporting information may be found in the online version of this article at the publisher's web‐site.

Supplementary Information
