# Supplementary material for: The BioFIND study: Characteristics of a clinically typical Parkinson's disease biomarker cohort
Source: Mov Disord. 2016 Apr 26;31(6):924–32. doi: 10.1002/mds.26613 (PMC5021110; doi:10.1002/mds.26613)
Supplement: Supplementary file 1 — Supplementary Information [file MDS-31-924-s001.docx]

**Supplemental materials**

**Table S1. Referral Sources**

|  | **PD** | **HC** |
| --- | --- | --- |
| BioFIND Study sites | 55.7% | 23.9% |
| MJFF communication | 12.2% | 8.0% |
| Specialists | 7.0% | 1.1% |
| Newspapers and magazines | 6.1% | 2.3% |
| Events | 2.6% | 2.3% |
| Advocacy | 1.7% | 2.3% |
| BioFIND Website | 0.9% | 26.1.% |
| Family or friend | 2.6% | 19.3% |

**Supplementary Methods: Biospecimen Collection**

The BioFIND study Biological Laboratory Manual is available online (<https://www.michaeljfox.org/page.html?biofind-clinical-study>) and summarized in this document.

*Blood Collection:* Blood is collected between 8am and 10am local time, ideally fasting, or, if not possible, on a low fat diet. Whole blood is collected in Sodium citrate, at room temperature, for PT/PTT and in EDTA for CBC and platelets (local lab, prior to lumbar puncture). Blood is also collected for DNA extraction in a tube containing Acid Citrate Dextrose (ACD) solution and shipped to the Repository within 5 days of blood draw, at room temperature. Additionally, whole blood is collected for RNA extraction, via PAXgene™ tubes. These tubes are the first drawn at phlebotomy, kept at the site at room temperature for 24 hours, then frozen at -80 degrees centrigrade, and subsequently, shipped to the Repository on dry ice.

*Plasma and Pellet Collection:* Whole blood is also collected for isolation of plasma and resultant pellet using an EDTA containing laboratory tube for initial collection. The 2ml micrcentrifuge aliquot tubes are pre-cooled by being placed on ice prior to procedure but after labeling. Within 30 minutes of blood collection, specimen is centrifuged at 4 degrees Centigrade for 15 minutes at 1500x g. Using a micropipette, 1.0ml of blood plasma (top layer) is aliquoted into 4-6 tubes. Great care is taken to not disrupt the pellet and to assure that the plasma is not contaminated by the pellet. Both the plasma aliquots and the pellet are frozen immediately following processing and transferred to a -80 degree freezer. Half (about three) of the plasma aliquots are shipped on dry ice to the NINDS Repository.

*CSF collection:* CSF is collected once on each participant, in the fasting state, or if fasting is not possible, on a low fat diet, between 8am and 10am local time. Approximately 15-20 cc of CSF is collected and then processed at room temperature, with a portion sent to the site clinical lab within 4 hours of collection. CSF is collected in a 50 ml conical tube, and then transferred to two 15 ml tubes, spun at 2000 x g for 10 minutes at room temperature, and aliquoted into 2 ml microcentrifuge tubes. The supernatant is then transferred to a -80 degree C freezer. Ten aliquots are shipped to the NINDS Repository.
